# Remembering Ad de Jong

**DOI:** 10.1016/j.mcpro.2023.100568

**Published:** 2023-06-03

**Authors:** Cécile A.C.M. van Els, Peter A. van Veelen, Albert J.R. Heck, Hugo D. Meiring

**Affiliations:** 1 Center for Infectious Disease Control, National Institute for Public Health and the Environment, Bilthoven, The Netherlands; 2 Center for Proteomics and Metabolomics, Leiden University Medical Center, Leiden, The Netherlands; 3 Biomolecular Mass Spectrometry and Proteomics, Utrecht University, Utrecht, The Netherlands; 4 Intravacc, Bilthoven, The Netherlands

It is with great sadness that we learned of the passing away of Ad P.J.M. de Jong, retired Principal Scientist at the National Institute for Public Health and the Environment (RIVM) and the National Vaccine Institute, both in Bilthoven (NL), in January, at the age of 75 years. Most Mass Spectrometrists do know him as a scientist who pioneered Biomolecular Mass Spectrometry already in the early 1990s, with a strong focus on developing *nano*scale Liquid Chromatography. Many research groups have copied his *nano*scale LC design, essentially based on the “Deans switching system,” which is still used in many research institutes worldwide (1). He had many collaborators in a large number of research groups. Together with immunologist Cécile van Els (from RIVM and Utrecht University, NL), he was a frontrunner in the field of ImmunoProteomics and ImmunoPeptidomics (2, 3, 4).

Although his father and mother wished a career in farming for him, Ad was trained as a chemical scientist at the University of Technology (Eindhoven, NL). He had a creative mind and was driven by curiosity and passion for his work. In 1977, he was appointed as Department Head of the Laboratory of Mass Spectrometry at the Vrije Universiteit (Amsterdam, NL) where he obtained his PhD degree in clinical chemistry in 1988. As Department Head of Molecular Spectroscopy at the Laboratory of Organic Analytical Chemistry at RIVM (Bilthoven, Netherlands), he worked on developing and applying MS-based techniques in environmental applications (with a strong focus on dioxin pollution in milk). He was always eager to take up something new and challenging. In the mid-1990s, immunologists in the Vaccines Sector of his institute reached out with an immunological project. They wanted to discover pathogen-derived peptides that are presented in so-called Major Histocompatibility Complex (MHC) molecules on cells during infections. Such peptide–MHC complexes can trigger T cell–mediated immune responses in fighting viral or bacterial infections. To learn the latest in analytical chemistry in this research field, Ad and one of his group members Jan ten Hove, went to Donald Hunt at the University of Virginia (USA), who had pioneered *nano*scale LC Electrospray Ionization MS for the identification of T cell epitopes and laid down the foundation of the field of ImmunoProteomics/Peptidomics. As an early adopter of Don’s method, Ad was one of the first to successfully identify non-self viral or bacterial epitopes on antigen presenting cells, typically present at sub-femtomolar range in peptide eluates from billions of cultured cells. Because of the analytical challenge to identify low abundant peptide species in chemically highly similar MHC–derived peptide mixtures, Ad kept thinking out of the box and designed novel separation systems. To tease these peptides apart prior to their MS analysis, he invented miniaturized separation strategies, using new types of offline and online fractionation strategies, employing columns of dazzling lengths (≥200 cm), miniaturized diameters (down to 25 μm), and mixed-bed or multi-bed stationary phases already in the mid-1990s (5, 6, 7). “We could always find him with a big smile sitting behind a Mass Spectrometer rather than behind his office desk, until the late/early hours, testing one of his new ideas on *nano*scale LC-MS for better performance,” immunologist and close colleague Cécile van Els remembers. Friend and colleague Peter van Veelen from Leiden University Medical Centre (Leiden, NL), who still saw him biweekly to cook and play chess together, adds: “Ad has been pioneering, teaching and spreading *nano*scale LC and ImmunoProteomics/Peptidomics *avant lettre* in the Netherlands and far beyond”. This was also the trigger for him to start *NanoSeparations*, one of the first commercial companies worldwide, providing a portfolio on tailor-made solutions and support on *nano*scale separation systems with a focus on biomolecular MS-based applications. With new generations of MS instruments coming on the market, gaining higher sensitivity, resolution, and acquisition, speed in ImmunoProteomics/Peptidomics seemed evident, but it was not. Without his tailor-made 2D pre-fractionation and *nano*scale LC separation, undersampling masked the rare epitopes of interest. Ad was very instrumental. Albert Heck (Utrecht University, NL) recalls the many demo activities with Ad when going for investments in new mass spectrometers to the demo sites of the manufacturers: “We did always try to bring, to my great pleasure, Ad and his *nano*scale LC system with us. His separation system by far outperformed those available in the demo labs. After such demos, manufacturers often asked if they could keep Ad’s *nano*scale LC system, to revisit their “failed” demos of the last couple of months and Ad also helped them”. Ad always kept helping people, with his love for *nano*scale LC, mass spectrometry, and immunoproteomics/peptidomics. Heck also remembers his very clever use of stable isotopically labeled pathogens to unambiguously discriminate between pathogenic-derived HLA peptides from self-peptides, a few years even before SILAC was born (8). “Ad was a very creative scientist and should be credited for quite a few inventions in our field, but he always liked to stay out of the spotlights and, frankly, I believe he did not like to write and publish” Albert recalls. Part of the technical developments and improvements were performed during his 6-months sabbatical leave in the early 2000s, that he spent at the Matthias Mann group at the University of Southern Denmark (Odense, DK). Together with Hugo Meiring, his right and “validating” hand in the group, Ad visited other (Immuno)Proteomics groups or Mass Spectrometer developers throughout Europe to exchange knowledge and plug in and play their *nano*separation system, always demonstrating superior performance.

Ad was a modest scientist, claiming little fame in his field. He was driven by his passion for mass spectrometry and by inventiveness, always chasing freedom and autonomy in his work. He kept his focus on pathogen-related proteomics analyses in vaccinology. Nowadays, the ImmunoProteomics/Peptidomics field has grown and moved into the elucidation of *neo*epitopes in cancer. Directly or indirectly, Ad did inspire many of these groups through his *nano*scale-LC approach in Mass Spectrometric analysis of ImmunoPeptidomes.

**Fig. 1:**
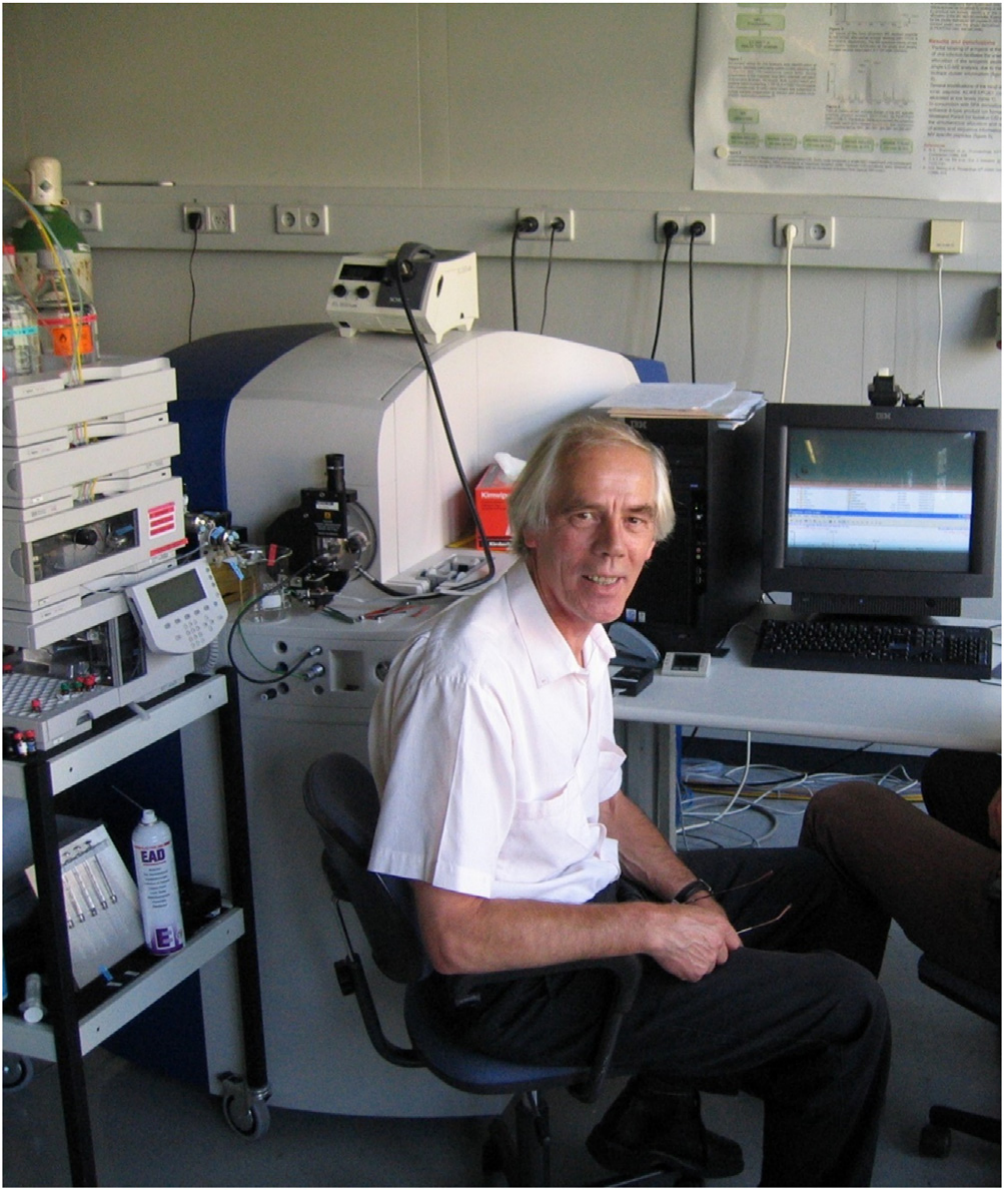
**Ad de Jong in 2005**.

Ad was married to Jeanne Beenackers, an autodidact artist and the love of his life. They got two daughters, Charlotte and Marloes, and two grandchildren, Anna and Max. They all loved the beauty of nature, in particular, rugged mountain regions and the sea: always enjoying hiking, bicycling, and sailing. Unfortunately, Jeanne passed away already in 2012 and in this same period, Ad developed cancer. This, all, coincided with his retirement. Missing both his beloved wife and work felt heavy on him. He never regained his good health. Nevertheless, he enjoyed being with family and good friends, as well as stimulating culture in his municipality and organizing art exhibitions of Jeanne’s work for many years, until he passed away at home on January 22, 2023. He will be dearly missed (Fig. 1).
